# Entanglement dynamics of Nitrogen-vacancy centers spin ensembles coupled to a superconducting resonator

**DOI:** 10.1038/srep21775

**Published:** 2016-02-23

**Authors:** Yimin Liu, Jiabin You, Qizhe Hou

**Affiliations:** 1 Department of Physics, Shaoguan University, Shaoguan, Guangdong 512005, China; 2 Department of Physics, The University of Hong Kong, Pokfulam Road, Hong Kong, China; 3 National Laboratory of Solid State Microstructures and School of Physics, Nanjing University, Nanjing 210093, China

## Abstract

Exploration of macroscopic quantum entanglement is of great interest in both fundamental science and practical application. We investigate a hybrid quantum system that consists of two nitrogen-vacancy centers ensembles (NVE) coupled to a superconducting coplanar waveguide resonator (CPWR). The collective magnetic coupling between the NVE and the CPWR is employed to generate macroscopic entanglement between the NVEs, where the CPWR acts as the quantum bus. We find that, this NVE-CPWR hybrid system behaves as a system of three coupled harmonic oscillators, and the excitation prepared initially in the CPWR can be distributed into these two NVEs. In the nondissipative case, the entanglement of NVEs oscillates periodically and the maximal entanglement always keeps unity if the CPWR is initially prepared in the odd coherent state. Considering the dissipative effect from the CPWR and NVEs, the amount of entanglement between these two NVEs strongly depends on the initial state of the CPWR, and the maximal entanglement can be tuned by adjusting the initial states of the total system. The experimental feasibility and challenge with currently available technology are discussed.

Recently, significant progress has been made in the quantum hybrid system consisting of a variety of physical systems, which combines the merits of two or more physical systems and mitigates their individual weaknesses. Especially, the hybrid quantum model including solid-state spin systems and superconducting coplanar waveguide resonator (CPWR) systems^1^,^2^,^3^,^4^,^5^,^6^,^7^,^8^,^9^,^10^,^11^, provides a promising platform to study the intriguing quantum optic phenomena as well as the fundamental quantum information (QI) science. Especially, spin-qubit in the solid-state system attract considerable interest because they can be used to store and transfer the QI^12^. Additionally, the coherence times of isolated or peculiar spins are usually long due to their weak interaction with the environment. For instance, the diamond nitrogen-vacancy (NV) centers, which are formed by nitrogen atoms substituting for carbon atoms and adjacent vacancies in a diamond, feature long coherence times of electron (nuclear) spin with about one *ms* (one *sec*) in a wide temperature range^13^,^14^,^15^,^16^,^17^,^18^,^19^,^20^,^21^,^22^,^23^,^24^,^25^,^26^,^27^. More importantly, the NV centers have the ability to coherently couple to various external fields simultaneously, such as both optical and microwave fields^28^,^29^. However, induced by the vacuum fluctuations of the photons, individual NV center couples to the CPWR with a very weak strength far below the linewidth of CPWR with dozens of *kHz*^30^,^31^, which is unfavorable for the coherent exchange of QI. Whereas, the collective coupling strength between a NV centers spin ensemble (NVE) and the CPWR will be enhanced by the value of 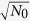 with 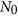 the total number of NV centers in the spin ensembles^2^,^3^,^32^. More importantly, compared to the conventional electric-dipole couplings mechanism, the employment of collective magnetic coupling for manipulating spin ensembles brings vital advantages^12^,^14^,^32^,^33^,^34^. To date, series of experimental demonstrations of strong magnetic couplings between NVE and CPWR^1^,^2^,^3^,^4^,^5^ have attracted considerable interest in the potential applications^35^,^36^,^37^,^38^,^39^,^40^,^41^.

On the other hand, due to the fragile nature of single-particle suffering severely from decoherence, it is desired to explore various channels to effectively construct highly entangled states in larger quantum systems, which is one of the central ingredients for large-scale quantum computation^42^. Recently, much particular attention has been paid to quantum entanglement of macroscopic samples^43^,^44^,^45^,^46^ owing to their unique quantum characteristics^47^,^48^, such as robustness to single-particle decoherence and relatively simple experimental realization. Consequently, developing experiments and theories for the useful interfacing of disparate macroscopic quantum systems like NVEs is increasingly important and interesting. Lately, many efforts have been devoted to the achievement of entanglement between separate macroscopic atomic ensembles, polar molecule ensembles, and electronic spin ensembles using different methods, including projective measurements^45^,^49^,^50^, quantum reservoir engineering^51^,^52^, spontanous/stimulated Raman scattering^53^,^54^, adiabatic quantum feedback^55^, intracavity electromagnetically induced transparency^48^, and so on.

In this work, we investigate a hybrid quantum system that consists of two separated NVEs coupled to a common CPWR, where each pair of NVE-CPWR interaction actually is a coherent coupling between two harmonic oscillators or bosonic fields with a collectively enhanced strength proportional to 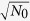. In our case, the collective NVE-CPWR magnetic coupling is used to generate entanglement between the NVEs, and decoherence effects from both the CPWR and the NVEs on the entanglement dynamics of NVEs have also been studied by employing the quantum trajectory method^56^,^57^,^58^,^59^. More importantly, we propose a practical scalable and tunable architecture in this model for investigating quantum dynamics of the NVEs and realizing entangled states between the NVEs. Furthermore, the present method provides us the potential feasibility of generating multi-NVE entanglement, which is a crucial element in the NVE-based quantum network. We find that, this NVE-CPWR hybrid system behaves as a system of three coupled harmonic oscillators, and the excitation prepared initially in the CPWR can be transferred and distributed in these two NVEs. In the nondissipative case, the entanglement of NVEs oscillates periodically and the maximal entanglement always keeps unity if the CPWR is initially prepared in the odd coherent state, and the situation becomes different in the case of even coherent state. Considering the dissipative effect from the CPWR and NVEs, the amount of entanglement between the two NVEs strongly depends on the initial state of the CPWR, and the maximal entanglement can be tuned by adjusting the initial states of system. Our further study reveals that the maximal entanglement between the NVEs could be achieved through accurately adjusting the tunable parameters, such as the initial states of the resonator field as well as the coupling rates. Our detailed analysis could find a way to extract the optimal experimental parameters for maximal entanglement between the NVEs using the increasingly-developed nanoscale solid-state technology, even in the presence of dissipative effects of the spin ensemble and superconducting resonator.

## Results

### System and Model

**Figure 1:**
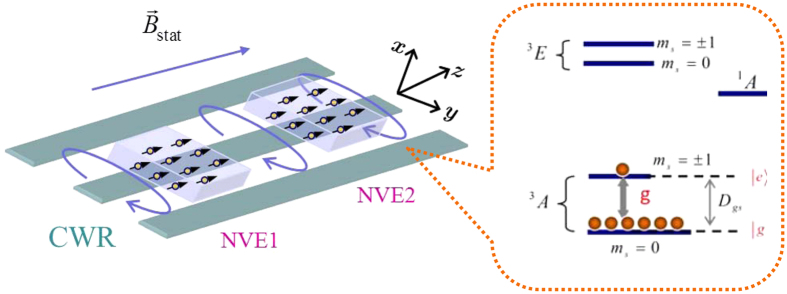
Schematic setup of the hybrid system consisting of two NVEs and a CPWR, where the two separated NVEs are placed in the resonator’s surface, and are coupled to the same CPWR through the collective magnetical coupling. The inset shows the level structure of single NV center, where the electronic ground and first excited states are electron spin triplet states with S = 1, and 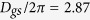 *GHz* is the zero-field splitting between the 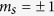 sublevels and the lowest energy 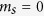 sublevel.

The system under consideration is illustrated in Fig. 1, the device we study is a combined NVE-CPWR system governed by the Hamiltonian


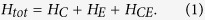


The microwave-driven CPWR (with the length *L*, the capacitance *C*_*c*_, and the inductance *F*_*c*_) consists of a narrow center conductor and two nearby lateral ground planes, whose Hamiltonian has the following form (in units of 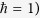 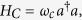 where *a* 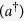 is the annihilation (creation) operator of the full-wave mode, and 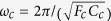 is the corresponding eigenfrequency. The distributions of current and voltage inside the CPWR have the expression 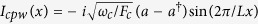 and 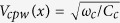

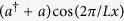
.

The Hamiltonian of a NVE containing 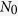 NV centers reads 
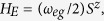
 where 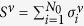 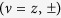 is the collective spin operator for the spin ensemble with 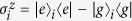, 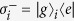 and 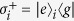. For clarity of our discussion, two symmetric Dicke excitation states 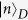 with 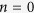 and 1 are introduced as 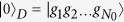 and 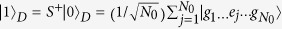, which could be encoded as the qubit of NVE. Through the collective magnetic-dipole coupling, the NVE-CPWR interaction Hamiltonian can be described by 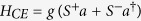 with *g* being the single NV vacuum Rabi frequency. Due to the fact that the mode wavelength of CPWR is larger than the spatial dimension of the NVE when the spin ensemble is placed near the field antinode, all the NV spins in ensemble interact symmetrically with a single mode of electromagnetic field. Using the Holstein-Primakoff (HP) transformation^60^,^61^, the spin operators can be mapped into the boson operators as follows: 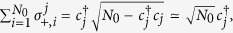 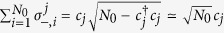, and 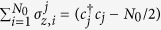, where the operators 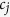 and 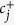 obey the standard boson commutator 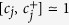 in the case of weak excitation.

So the total Hamiltonian of the NVE-CPWR coupling system is given by


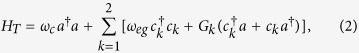


where 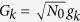 represents the collective coupling strength between the *k*-th NVE and CPWR with 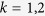. Meeting the condition 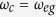, we can obtain the following Hamiltonian 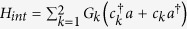. One can find that the interactions between the two NVEs and the CPWR could be reduced to the coupling of three bosonic fields or harmonic oscillators. Taking the dissipative effects from the NVEs and the CPWR into account, the dissipative dynamics of the total system can be effectively described by employing the quantum trajectory method^56^,^57^ with the conditional Hamiltonian^58^


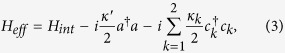


where 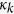 and 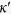 are the decay rates of the *k*-th NVEs and the CPWR, respectively. This is a reasonable assumption for the region of interest, where the decay rates are not dominant, and the CPWR has a very small probability to be detected with a photon. For simplicity, here we have assumed 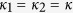 in our model.

### Entanglement dynamics of Nitrogen-vacancy centers spin ensembles

In this section we will focus on the Entanglement dynamics of Nitrogen-vacancy centers spin ensembles. According to the Heisenberg motion equations, we can obtain the differential equations of the operators 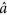 and 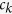 as


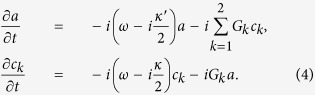


Considering the initial conditions 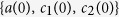, we obtain the following analytical solution


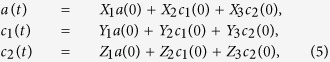


where the expression of coefficients *X*_*i*_, *Y*_*i*_, and *Z*_*i*_ (*i* = 1, 2, 3) are 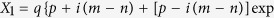 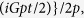

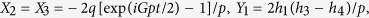
 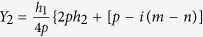

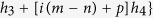
,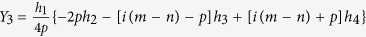, and 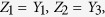 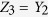 with 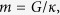 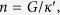 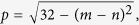 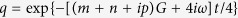, 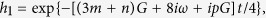 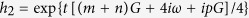, 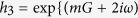 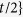 and 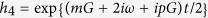. Here we set 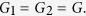


Suppose that the CPWR is initially prepared in an arbitrary normalized superposition of the coherent state 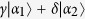
62,63 and the NVEs are prepared in their vacuum states 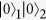, where 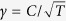, 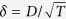, and 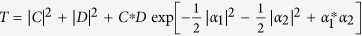

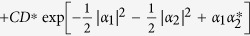
 are the normalized coefficient with 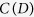 arbitrary complex numbers and * complex conjugation. Under these initial conditions, the time-dependent wave function of the total system 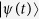 can be expressed as 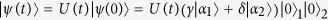 with time evolution operator 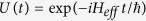. A straightforward calculation yields


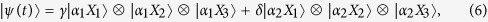


where we have used the relationships 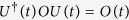 and 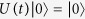. To investigate the entanglement dynamics between the NVEs, we need to trace over the degree of freedom of the CPWR as 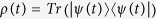, which yields


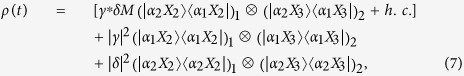


where *h.c.* denotes Hermitian conjugate and 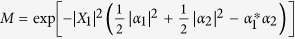 is the inner product of the two coherent states 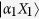 and 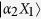.

Through the calculation, the concurrence of two NVEs has the form


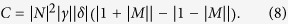


Here we have employed the relations 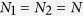, 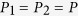, and 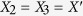.

Besides, the average phonon number 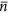 of the CPWR can also be obtained as follows


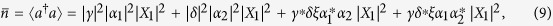


where 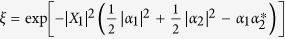.

Firstly, we consider the simple case that the CPWR is initially prepared in the odd coherent state 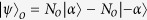 and the even coherent state 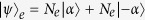 with normalization constants 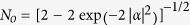 and 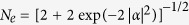, respectively. So we can obtain 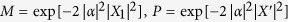 and 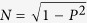. As a result, the concurrence of NVEs and the average phonon number of CPWR can be expressed by


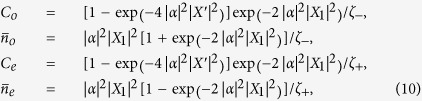


where 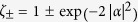.

**Figure 2:**
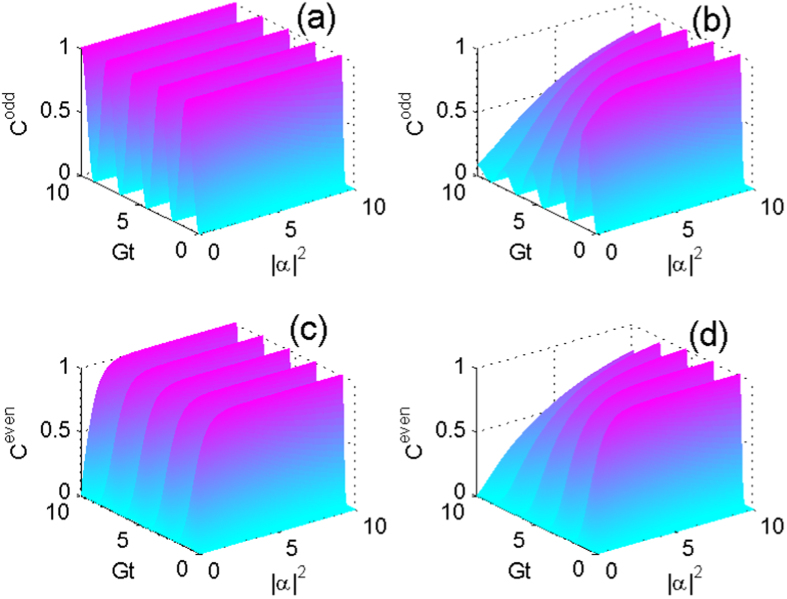
The concurrence as a function of *Gt* and 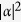 when the CPWR is initially prepared in (**a**,**b**) odd coherent state 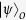 and (**c**,**d**) even coherent state 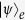, respectively. The left and right panels denote the nondissipative case 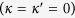, and the dissipative case 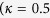, 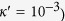, respectively.

The entanglement dynamics of NVEs is plotted in Fig. 2 as functions of time and parameter 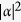 in the nondissipative/dissipative case. In the present system, the collective magnetic coupling between the NVE and the CPWR is employed to generate macroscopic entanglement between the spin ensembles, where the CPWR acts as the common quantum bus. One can find that, in the nondissipative case, the concurrence oscillates periodically and the maximal value of the concurrence 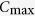 always keeps unity for any values of 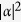 in the case of odd coherent state 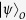 in Fig. 2(a). It implies that the excitation initially prepared in CPWR is reversibly transferred between the NVEs and the CPWR. The situation becomes different, as shown in the Fig. 2(c) for the even coherent state 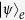, and the maximal values 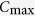 are gradually close to one with the growth of the 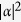. Considering the dissipative effect from the CPWR and NVEs in Fig. 2(b,d), it is worth noting that the amount of entanglement between the two NVEs strongly depends on the initial state of the CPWR, and 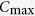 increases gradually. Another interesting feature is that the values of 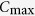 are enhanced with the growth of the values of 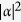. therefore, this NVE-CPWR hybrid system behaves as a composite system of three coupled harmonic oscillators, and the excitation can be transferred and distributed in these two NVEs if we initially prepare the excitation in the common databus, namely, the CPWR.

**Figure 3:**
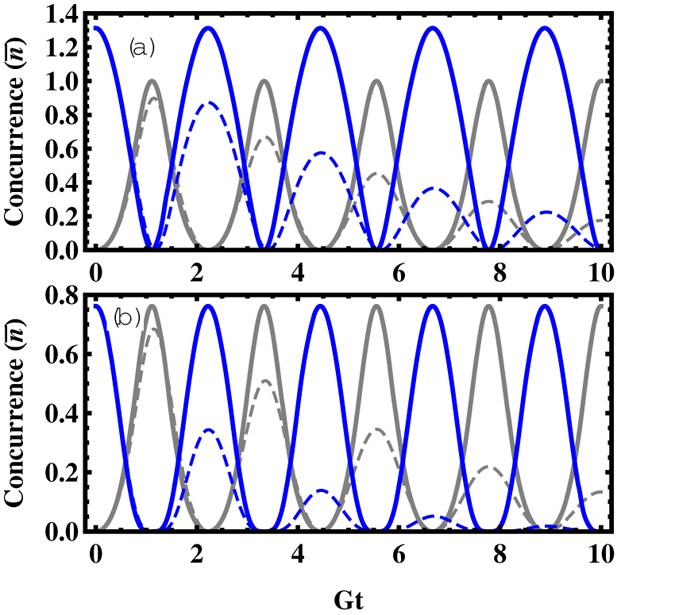
The concurrence (the grey line) and the average phonon number (the blue line) as functions of the dimensionless time, where we set 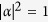, and the CPWR is initially prepared in the odd coherent state (**a**), and even coherent state (**b**), respectively. The solid and dashed lines represent the nondissipative case 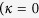, 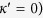 and the dissipative case 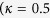, 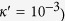, respectively.

**Figure 4:**
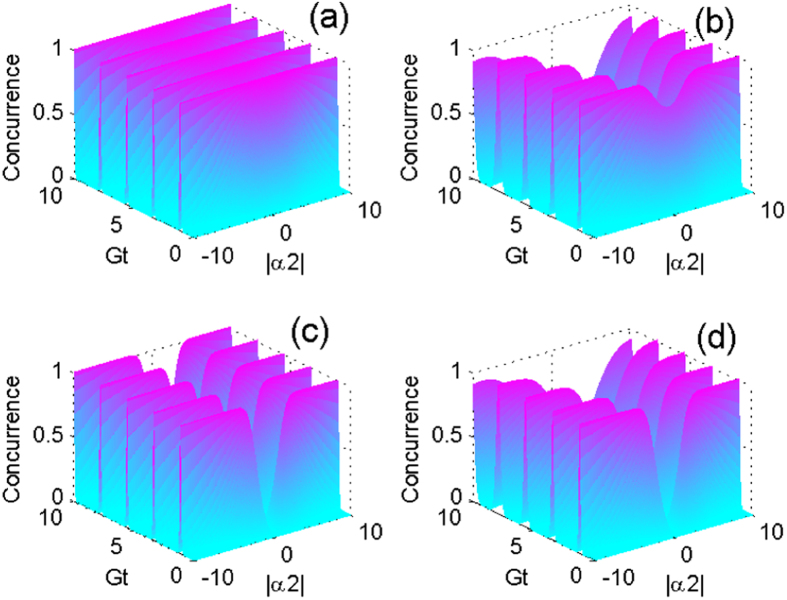
The concurrence as a function of *Gt* and 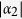 when the CPWR is initially prepared in the coherent superposition state (**a**,**b**) 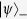 and (**c**,**d**) 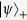, respectively, where 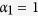. The left and right panels denote the nondissipative case 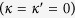, and the dissipative case 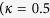, 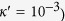, respectively.

In Fig. 3, we explicitly quantify the time-dependent concurrence and the average number of phonon 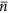 of the CPWR by setting 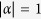. One can find that the maximal values of concurrence appear if and only if 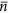 is zero in the case of odd or even coherent state. However, the maximal values of concurrence can reach one in the case of the odd coherent state, rather than the even coherent state, which could be understood by the above expression in Eq. (10). We also show that the concurrence and 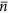 oscillate with the same period, and the period of the odd coherent state 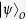 is longer than that of the even coherent state 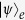. To study the dynamics in more general cases, we assume that the CPWR is initially prepared in a coherent superposition state 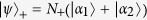 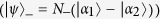 with 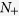 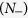 the normalized coefficient. The dynamics of concurrence is plotted as functions of time and parameter 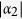 in Fig. 4, where we set 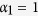. It turns out that the of two NVEs in this case are entangled in an oscillating way, and the decoherence effects degrade the entanglement between the spin ensembles, as shown in Fig. 4(b,d).

## Discussion

We now survey the relevant experimental parameters. First, a full wave frequency 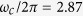 GHz of resonator mode could be obtained if the CPWR has the inductance 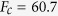 nH and the capacitance 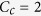 pF. Second, to ensure that the NVE-CPWR coupling could obtain the maximal values, the NVEs should be located symmetrically in the position where the magnetic field of resonator is maximal. Thirdly, the feasibility of our scheme could be confirmed by series of experimental demonstration of NVE-CPWR strong magnetic coupling with the strength ~ dozens of MHz, as well as the experimental advances in excellent quantum control in the quantum hybrid system consisting of a superconducting flux qubit and NVE. Finally, the electron relaxation time 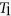 of NV centers could reach 6 ms at room temperature^64^, even reach 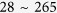 s if we place NV centers at lower temperature^65^. In addition, using a spin echo sequence, the dephasing time can be greatly enhanced by decoupling the electron spin from its local environment. Based this technique, the dephasing time of the NVE reaches 3.7 *μ*s at room temperature^35^, and the dephasing time 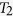 for NVE with natural abundance of ^13^*C* has been reported that it could reach 0.6 *ms*^66^, which has been prolonged to be *T*_2_ = 1.8 *ms* in the isotopically pure diamond sample^67^. Another major decoherence source is the dipole-interaction between the NV center spins and the redundant Nitrogen spins, which could be suppressed by enhancing the conversion rate from nitrogen to NV, while keeping the almost stable collective coupling constants^35^. Alternatively, by applying the external driving field to the electron spins of the Nitrogen atoms, the coherence time of the NVE could be It would increased if the flip-flop processes is much slower than the rate of flipping of these spins^68^.

In the above discussion, the the detrimental influence from the nuclear spin, such as ^13^*C* defects in the spin ensemble have been ignored, nevertheless, this problem could be alleviated by isotopically purified ^12^*C* diamond through the purification technique^67^,^68^. Note that the present method provides us the potential feasibility of generating multi-NVE entanglement, which is a crucial element in the NVE-based scalable quantum network. We emphasize that the multi-NVE dynamics itself is more complicated and could exhibit richer dynamical behavior than the two-qubit case^69^,^70^,^71^. Therefore, it is desirable to investigate the quantum dynamics of NVEs in a scalable way, and to develop efficient methods for controlling the entanglement dynamics of many NVEs in a common resonator. However, this issue goes beyond the scope of the present paper. Noticeably, the multi-NVE dynamics in different model have been studied for large-scale arrays^72^.

In the following we provide the reason that we can use the quantum jump model (Eq. (3)) to study the dephasing effect. In this work we give a phenomenological model for deeply understanding the decay of NVE collective excitation induced by the dephasing effects, which is mainly from the inhomogeneous broadening. In other word the dephasing time of the NVE is around 1/*κ*. Because of the local environment difference, the frequencies of the electron spins in NVE are not identical, and the mean frequency is denoted as 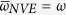. For single excitation of NVE, if the initial state is 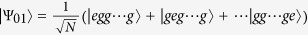, we will lose the information of the collective excitation due to the inhomogeneous broadening. Therefore, after dephasing time 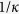, the NVE will reach the final state 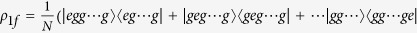, which is completely mixed state with excitation number 1. We can easily to verify that the overlap between initial and the final state 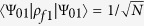 approaches to zero. Therefore, the initial and final state can be viewed as two orthogonal states. Besides, we find that the final mixed state 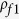 is nearly decoupled with the reaonator mode, as there is no collective coupling enhancement for the mixed state. The single excitation decay equation can be written as 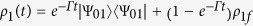. For the higher excitation states (Dicke states) with low excitation number 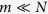, the similar results are held too. The initial collective state 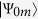 will decay to the final mixed state 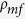 because of dephasing, and the excitation number *m* is fixed during the processing. The initial and the final states for the *m*-th excitation states are also orthogonal. The evolution equation of it is 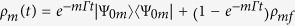. Therefore, we find that the pure dephasing induces the amplitude decay of the collective excitation of NVE. The decay rate is equal to the dephasing rate of the NVE.

In order to measure the macroscopic entanglement in realistic experimetns, we need to transfer the state from the NVEs to the states of two additional small flux qubits, each of which is attached on a NVE. So the task of entanglement dectection can be performed by the direct measurement on the states of additional flux qubits, and implementation of transferring the state from NVEs to flux qubits could be realized by using the SWAP gate between the *j*-th NVE and the *j*-th flux qubits, like the method in^40^. We should note that, in order to guarantee the collective mode detected by the small qubit is the same mode prepared by the resonator, the coupling strength between the each NV center to the resonator mode should be proportional to that to the small qubit^40^.

In summary, we have presented a study on the dynamics of entanglement between spin ensembles via the collective coupling between the CPWR and NVEs in such a hybrid system composed by a CPWR and two NVEs. This NVE-CPWR hybrid system behaves as a system of coupled harmonic oscillators, and the excitation prepared initially in the CPWR can be transferred and distributed in these two NVEs, where the CPWR plays the role of common databus. The decoherence effects from the CPWR and NVEs on the quantum dynamics of the entanglement between spin ensembles have also been studied. Therefore, the present system provides a platform to generate quantum entanglement between two or more NVEs embedded in the same resonator, which may be another route toward building a distributed QIP architecture and future NVE-based quantum network.

## Method

### Calculations of concurrence

Before using the concept of concurrence for bipartite entangled nonorthogonal states^73^,^74^ to measure entanglement between the NVEs, we transform the nonorthogonal form in Eq.(7) into an orthogonal form by rebuilding two orthogonal and normalized states as basis of the two-dimensional Hilbert space. Using the Gram–Schmidt orthogonalization process^75^, we can define


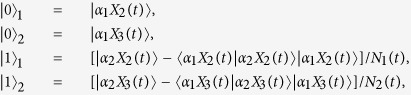


where 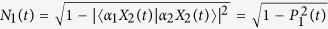 and 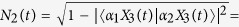

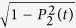
. In this new basis, the reduced density operator of NVEs (Eq.(7)) can be rewritten into


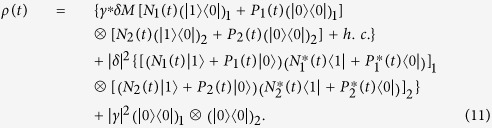


Therefore the elements of the orthogonal form *ρ* are


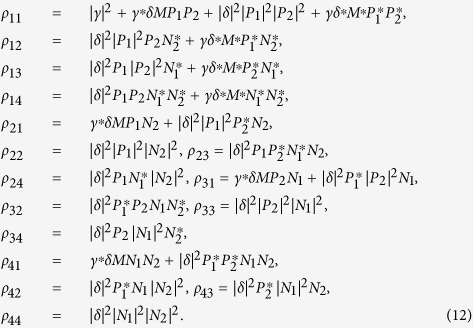


It is easy to obtain the square roots of eigenvalues of the matrix 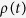 in Eq. (11) as 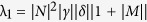, 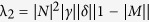, and 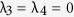. As a result, the concurrence of two NVEs has the form


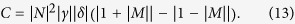


## Additional Information

**How to cite this article**: Liu, Y. *et al.* Entanglement dynamics of Nitrogen-vacancy centers spin ensembles coupled to a superconducting resonator. *Sci. Rep.* **6**, 21775; doi: 10.1038/srep21775 (2016).
